# Identification of Common CD8^+^ T Cell Epitopes from Lassa Fever Survivors in Nigeria and Sierra Leone

**DOI:** 10.1128/JVI.00153-20

**Published:** 2020-06-01

**Authors:** Saori Sakabe, Jessica N. Hartnett, Nhi Ngo, Augustine Goba, Mambu Momoh, John Demby Sandi, Lansana Kanneh, Beatrice Cubitt, Selma D. Garcia, Brian C. Ware, Dylan Kotliar, Refugio Robles-Sikisaka, Karthik Gangavarapu, Luis M. Branco, Philomena Eromon, Ikponmwosa Odia, Ephraim Ogbaini-Emovon, Onikepe Folarin, Sylvanus Okogbenin, Peter O. Okokhere, Christian Happi, Pardis C. Sabeti, Kristian G. Andersen, Robert F. Garry, Juan Carlos de la Torre, Donald S. Grant, John S. Schieffelin, Michael B. A. Oldstone, Brian M. Sullivan

**Affiliations:** a Viral Immunobiology Laboratory, Department of Immunology and Microbiology, The Scripps Research Institute, La Jolla, California, USA; b Department of Microbiology and Immunology, Tulane University School of Medicine, New Orleans, Louisiana, USA; c Viral Hemorrhagic Fever Program, Kenema Government Hospital, Kenema, Sierra Leone; d Ministry of Health and Sanitation, Freetown, Sierra Leone; e Eastern Polytechnic Institute, Kenema, Sierra Leone; f Njala University, Moyamba, Sierra Leone; g FAS Center for Systems Biology, Harvard University, Broad Institute of MIT and Harvard, Cambridge, Massachusetts, USA; h Scripps Translational Research Institute, The Scripps Research Institute, La Jolla, California, USA; i Department of Molecular and Experimental Medicine, The Scripps Research Institute, La Jolla, California, USA; j Zalgen Labs, Germantown, Maryland, USA; k African Center of Excellence for Genomics of Infectious Disease (ACEGID), Redeemers University, Ede, Nigeria; l Institute of Lassa Fever Research and Control, Irrua Specialist Teaching Hospital, Irrua, Nigeria; m Department of Biological Sciences, Redeemers University, Ede, Nigeria; n Department of Medicine, Irrua Specialist Teaching Hospital, Irrua, Nigeria; o Department of Medicine, Faculty of Clinical Sciences, Ambrose Alli University, Ekpoma, Nigeria; p Sherlock Biosciences, Cambridge, Massachusetts, USA; q Danaher Corporation, Washington, DC, USA; r College of Medicine and Allied Health Sciences, University of Sierra Leone, Freetown, Sierra Leone; s Department of Pediatrics, Tulane University School of Medicine, New Orleans, Louisiana, USA; University of North Carolina at Chapel Hill

**Keywords:** epitopes, Lassa fever, Lassa virus, memory T cells, arenavirus

## Abstract

Early and robust T cell responses have been associated with survival from Lassa fever (LF), but the Lassa virus-specific memory responses have not been well characterized. Regions within the virus surface glycoprotein (GPC) and nucleoprotein (NP) are the main targets of the Lassa virus-specific T cell responses, but, to date, only a few T cell epitopes within these proteins have been identified. We identified GPC and NP regions containing T cell epitopes and HLA haplotypes from LF survivors and used predictive HLA-binding algorithms to identify putative epitopes, which were then experimentally tested using autologous survivor samples. We identified 12 CD8-positive (CD8^+^) T cell epitopes, including epitopes common to both Nigerian and Sierra Leonean survivors. These data should be useful for the identification of dominant Lassa virus-specific T cell responses in Lassa fever survivors and vaccinated individuals as well as for designing vaccines that elicit cell-mediated immunity.

**IMPORTANCE** The high morbidity and mortality associated with clinical cases of Lassa fever, together with the lack of licensed vaccines and limited and partially effective interventions, make Lassa virus (LASV) an important health concern in its regions of endemicity in West Africa. Previous infection with LASV protects from disease after subsequent exposure, providing a framework for designing vaccines to elicit similar protective immunity. Multiple major lineages of LASV circulate in West Africa, and therefore, ideal vaccine candidates should elicit immunity to all lineages. We therefore sought to identify common T cell epitopes between Lassa fever survivors from Sierra Leone and Nigeria, where distinct lineages circulate. We identified three such epitopes derived from highly conserved regions within LASV proteins. In this process, we also identified nine other T cell epitopes. These data should help in the design of an effective pan-LASV vaccine.

## Abstract (precis)

The high morbidity and mortality associated with clinical cases of Lassa fever, together with the lack of licensed vaccines and limited and partially effective interventions, make Lassa virus (LASV) an important health concern in its regions of endemicity in West Africa. Previous infection with LASV protects from disease after subsequent exposure, providing a framework for designing vaccines to elicit similar protective immunity. Multiple major lineages of LASV circulate in West Africa, and therefore, ideal vaccine candidates should elicit immunity to all lineages. We therefore sought to identify common T cell epitopes between Lassa fever survivors from Sierra Leone and Nigeria, where distinct lineages circulate. We identified three such epitopes derived from highly conserved regions within LASV proteins. In this process, we also identified nine other T cell epitopes. These data should help in the design of an effective pan-LASV vaccine.

## INTRODUCTION

Lassa fever (LF), a hemorrhagic illness caused by infection with the Old World mammarenavirus, Lassa virus (LASV), is an important health concern in its regions of endemicity of West Africa due to high morbidity and mortality among hospitalized patients. Limited health care infrastructure in rural West Africa makes the study of LF in humans difficult. This difficulty is compounded by the classification of LASV as a biosafety level 4 pathogen.

Despite the challenges in assessing immune responses during LF, evidence indicates that T cell responses are critical for control and clearance of acute LASV infection (1), with robust T cell responses observed in human and nonhuman primate LF survivors (2, 3). In contrast, antibody responses are slow to develop, with neutralizing antibodies detected only months after viremia has cleared (2, 4, 5). Despite their importance during acute infection, T cell responses to LASV infection in humans have been poorly characterized. A case study of an infected health care worker described robust CD4-positive (CD4^+^) and CD8^+^ T cell responses, a prolonged CD8^+^ T cell response, and a delayed LASV-specific IgG response (2). A separate study of eight LF survivors from the Republic of Guinea showed proliferative CD4^+^ T cell responses in response to a nucleoprotein (NP) antigen. Six CD4^+^ T cell epitopes with variable cross-reactivity to Lassa lineage-specific sequences were described for one of these patients (6).

Previous infection with LASV appears to protect against LF after reexposure to LASV, but the contribution of cell-mediated and humoral immunity to protection from subsequent exposure remains to be determined. A recent LASV challenge study in nonhuman primates (NHPs) found that NHPs vaccinated with a measles virus vector encoding both the glycoprotein (GPC) and NP induced nearly sterilizing immunity after a single dose, while control of viremia was not as effective when the NP was excluded (7). This finding is consistent with our observations that the majority of LASV survivors harbor T cells that respond to both NP and GPC (8).

Ideally, a LASV vaccine should protect against strains of all major lineages of LASV found in geographically distinct areas of West Africa. These lineages coincide with regional populations of Mastomys natalensis, the major natural reservoir of LASV (9–15). Previously, we documented that LF survivors with LASV-specific CD8^+^ T cell responses to antigens from one lineage respond to antigens from a different LASV lineage (8). We also found that specific regions of the LASV GPC and NP harbor the majority of T cell epitopes against LASV (8). Here, we identify peptide epitopes present within these regions and identify common T cell epitopes between Nigerian and Sierra Leonean LF survivors.

## RESULTS

To identify LASV-specific CD8 T cells, we created a library of recombinant single-cycle vesicular stomatitis virus (rscVSV) encoding LASV antigens (NP, GP1, and GP2) and 60-amino acid (aa) polypeptides (herein fragments) derived from these antigens (Table 1) from the lineage IV Josiah strain. We detected mRNA and protein expression in rscVSV-infected BHK-21 cells for NP and GP1, but only mRNA expression for GP2 and each of the ∼60-aa polypeptides. Importantly, we observed robust CD8^+^ T cell responses following infection of human peripheral blood mononuclear cells (PBMCs) with rscVSV-expressing antigens for which only mRNA was readily detected.

**TABLE 1:** Amino acid positions encoded by rscVSVs expressing LASV antigens

Antigen	AA position
GP1	1–279
GP2	214–491
GPC-f1	1–58
GPC-f2	34–93
GPC-f3	75–134
GPC-f4	115–174
GPC-f5	153–212
GPC-f6	194–259
GPC-f7	240–299
GPC-f8	284–343
GPC-f9	322–381
GPC-f10	359–418
GPC-f11	404–463
GPC-f12	445–491
NP	1–569
NP-f1	1–59
NP-f2	40–98
NP-f3	79–145
NP-f4	126–185
NP-f5	166–225
NP-f6	206–265
NP-f7	246–310
NP-f8	291–350
NP-f9	331–390
NP-f10	371–430
NP-f11	411–476
NP-f12	457–527
NP-f13	508–569

Using these reagents, we sought to identify LASV-specific CD8^+^ T cell responses to NP and GPC as well as responses to regions within these viral proteins to identify sequences that harbor T cell epitopes. We use antigens from lineage IV viruses which circulate in Sierra Leone to stimulate T cell responses from Sierra Leonean and Nigerian survivors, despite the latter likely being infected with lineage II and III LASV. We previously described results indicating comparable CD8^+^ T cell responses between Nigerian and Sierra Leonean LF survivors to lineage IV antigens (8).

**FIG 1:**
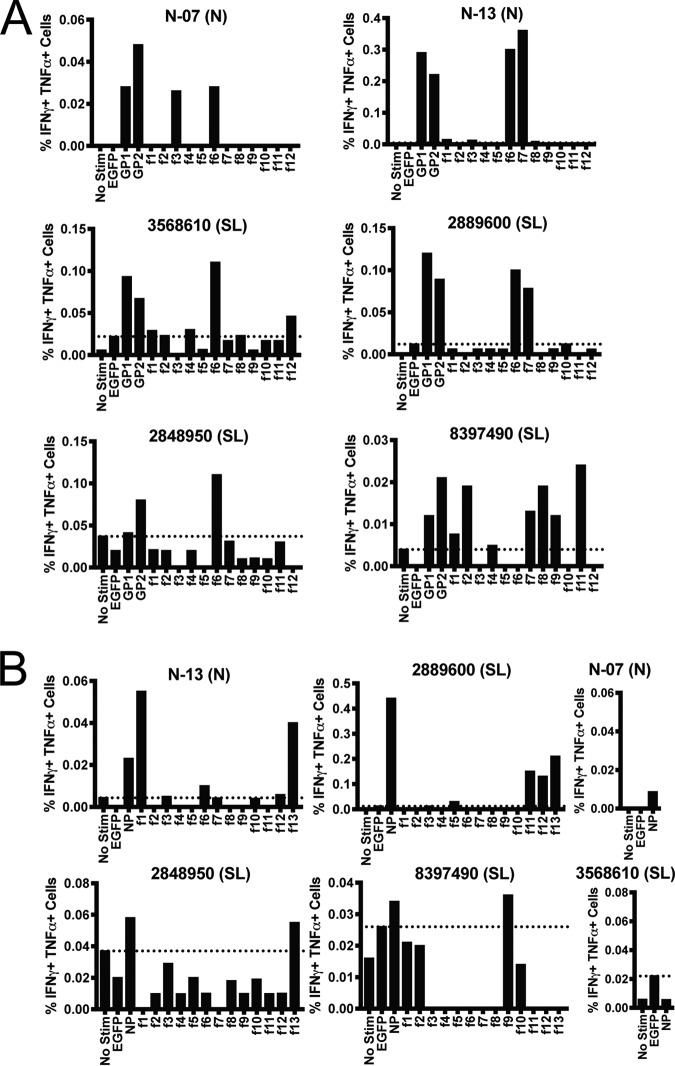
CD8^+^ T cell responses from LF survivors from Nigeria (N) and Sierra Leone (SL). (A) PBMCs were incubated with rscVSVs encoding EGFP (negative control), GP1, GP2, or ∼60-amino acid polypeptides derived from GPC sequences overnight in the presence of brefelin A. Cells were surface stained for CD3e and CD8 before fixation, permeabilization, and staining with antibodies against TNF-α and IFN-γ. Graphs indicate the percentage of CD3^+^ CD8^+^ cells positive for both TNF-α and IFN-γ by flow cytometry. Positive gates were set at a mean fluorescence intensity of >1.2 log_10_ over the median negative control, and dotted horizontal lines indicate levels of highest negative-control sample (no stim or enhanced green fluorescent protein [EGFP]). (B) Same as panel A except PBMCs were incubated with rscVSVs encoding NP and associated polypeptides. CD8^+^ T cell responses to NP using PBMCs from N-07 and 3568610 were considered negative, and responses to NP fragments were not assessed.

**FIG 2:**
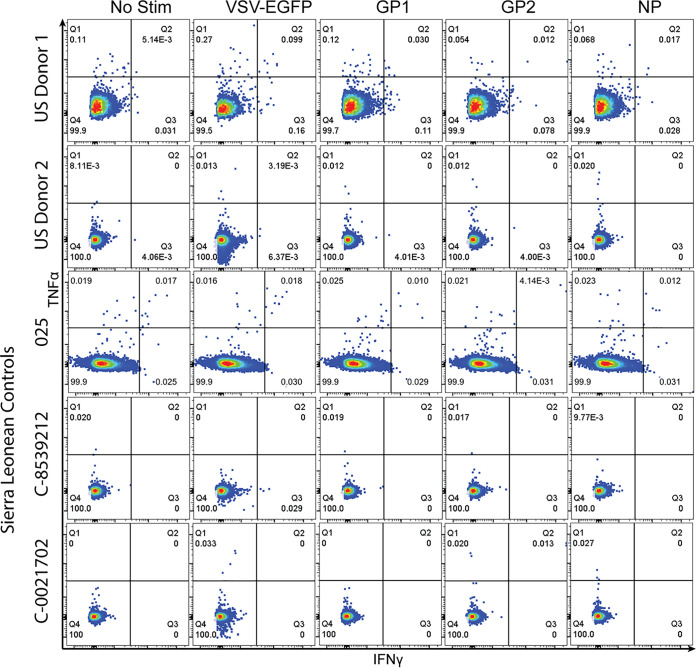
CD8^+^ T cell responses to rscVSV encoding EGFP and LASV antigens GP1, GP2, and NP, as well as unstimulated controls, were assessed in two U.S. donors with no history of travel to West Africa and three Sierra Leoneans seronegative for LASV.

PBMCs from Sierra Leonean and Nigerian LF survivors were incubated overnight with rscVSVs encoding GP1, GP2 (Fig. 1A), NP (Fig. 1B), and overlapping fragments from each antigen (Fig. 1) and assessed for intracellular expression of interferon gamma (IFN-γ) and tumor necrosis factor alpha (TNF-α). We observed no responses in two donors from the United States with no previous travel to Africa and in three Sierra Leonean individuals with no history of LF and who were seronegative at the time of blood collection (Fig. 2). We observed CD8^+^ T cell responses to rscVSVs encoding LASV GPC and the NP in four LF survivors (N-13, 2889600, 2848950, and 8397490), while we observed CD8^+^ T cell responses to rscVSVs encoding LASV GPC but not those encoding NP in LF survivors N-07 and 3568610 (Fig. 1). As there was no response to the NP, stimulations with rscVSVs encoding overlapping fragments derived from NP sequences were not performed.

We used information from T cell responses to overlapping fragments to deduce epitope-containing regions as described (8, 16). If we observed IFN-γ- and TNF-α-expressing CD8^+^ T cells in response to rscVSVs encoding two adjacent overlapping sequences, we deduced that the epitope-containing region (or deduced epitope) occurs in the overlapping sequence. If we observed IFN-γ- and TNF-α-expressing CD8^+^ T cells in response to one rscVSV and no response to rscVSVs encoding adjacent sequences, we deduced that the epitope-containing region occurs in the nonoverlapping region plus an additional seven amino acids in the overlapping regions on either side. These additional amino acids account for most epitopes that would have been only partially encoded by rscVSVs encoding adjacent sequences. Most deduced epitopes were different between Nigerian and Sierra Leonean survivors (8), but we identified individuals from both survivor groups that had T cell responses to common regions within the NP and GPC (Table 2).

**TABLE 2:** HLA (class I) profiles, deduced epitopes, and peptide sequences with top predicted restriction and percentile rank for each^*a*^

Subject (list of HLAs)	Deduced epitope	Peptide sequence	Peptide position	Predicted restriction	Percent rank
N-07	GPC 206-245	GNWDCIMTSY	GPC 208-217	A*2902	1.14
	GPC 206-245	WDCIMTSYQY	GPC 210-219	A*2902	0.94
(A*290201,	GPC 206-245	CIMTSYQYLI	GPC 212-221	B*5301	1.02
A*660101,	GPC 206-245	TWEDHCQFSR	GPC 226-235	C*0401	9.4
B*530101,	GPC 206-245	HCQFSRPSPI	GPC 230-239	A*6601	8.2
B*8101,	GPC 206-245	FSRPSPIGYL	GPC 233-242	C*1801	0.9
C*040101,	GPC 206-245	**RPSPIGYLGL**	**GPC 235-244**	B*8101	**0.03**
C*1801)					
N-13	GPC 240-259	YLGLLSQRTR	GPC 241-250	A*7401	1.6
	GPC 240-259	LSQRTRDIYI	GPC 245-254	B*5301	7.25
	GPC 240-259	**RTRDIYISRR**	**GPC 248-257**	A*7401	**0.09**
(A*330301,	NP 1-46	MSASKEIKSF	NP 1-10	C*0302	2.0
A*740101,	NP 1-46	ASKEIKSFLW	NP 3-12	B*5801	0.48
B*530101,	NP 1-46	IKSFLWTQSL	NP 7-16	C*0302	4.0
B*580101,	NP 1-46	SFLWTQSLRR	NP 9-18	A*3303	0.32
C*030202,	NP 1-46	KDAQALLHGL	NP 33-42	B*5801	2.85
C*030202)	NP 521-569	TPHCALMDCI	NP 526-535	B*5301	1.45
	NP 521-569	HCALMDCIMF	NP 528-537	B*5301	2.65
	NP 521-569	TSVLRAVLPR	NP 547-556	A*3303	0.94
	NP 521-569	**AVLPRDMVFR**	**NP 552-561**	A*7401	**0.08**
	NP 521-569	LPRDMVFRTS	NP 554-563	B*5301	3.1
	NP 521-569	DMVFRTSTPR	NP 557-566	A*3303	0.55
	NP 521-569	FRTSTPRVVL	NP 560-569	C*0302	2.3
3568610	GPC 206-246	CIMTSYQYLI	GPC 212-221	A*0201	2.3
	GPC 206-246	SYQYLIIQNT	GPC 216-225	A*2301	4.8
(A*020101,	GPC 206-246	QYLIIQNTTW	GPC 218-227	A*2301	0.35
A*230101,	GPC 206-246	**FSRPSPIGYL**	**GPC 233-242**	C*1601	**0.68**
B*070601,	GPC 206-246	**RPSPIGYLGL**	**GPC 235-244**	B*0706	**0.03**
B*450101,					
C*070201,					
C*160108)					
2889600	GPC 240-259	LSQRTRDIYI	GPC 245-254	A*3001	3.15
	GPC 240-259	**QRTRDIYISR**	**GPC 247-256**	A*3402	**0.59**
	GPC 240-259	**RTRDIYISRR**	**GPC 248-257**	A*3402	**0.55**
(A*300101,	GPC 240-259	RDIYISRRLL	GPC 250-259	B*0702	3.3
A*340201,	NP 424-569	ATQPGLTSAV	NP 440-449	C*1701	1.4
B*070201,	NP 424-569	**LPRNMVITCQ**	**NP 453-462**	B*0702	**2.7**
B*420101,	NP 424-569	KDIKLIDIAL	NP 477-486	A*3001	3.45
C*070201,	NP 424-569	IKLIDIALSK	NP 479-488	A*3402	0.46
C*170101)	NP 424-569	KYENAVWDQY	NP 493-502	B*0702	6.2
	NP 424-569	**HMHTGVVVEK**	**NP 507-516**	A*3402	**0.64**
	NP 424-569	TPHCALMDCI	NP 526-535	B*4201	0.95
	NP 424-569	TSVLRAVLPR	NP 547-556	A*3402	1.9
	NP 424-569	RAVLPRDMVF	NP 551-560	C*1701	2.5
	NP 424-569	AVLPRDMVFR	NP 552-561	A*3402	0.25
	NP 424-569	**LPRDMVFRTS**	**NP 554-563**	B*0702	**1.75**
	NP 424-569	DMVFRTSTPR	NP 557-566	A*3402	1.6
	NP 424-569	MVFRTSTPRV	NP 558-567	A*3001	1.53
	NP 424-569	FRTSTPRVVL	NP 560-569	C*0702	0.45
2848950	GPC 206-290	HCQFSRPSPI	GPC 230-239	C*1601	8.7
	GPC 206-290	CQFSRPSPIG	GPC 231-240	B*4901	32
	GPC 206-290	FSRPSPIGYL	GPC 233-242	C*1601	0.68
(A*340201,	GPC 206-290	SRPSPIGYLG	GPC 234-243	C*0701	6.0
A*740101,	GPC 206-290	YISRRLLGTF	GPC 253-262	A*3402	13
B*350101,	GPC 240-259	YLGLLSQRTR	GPC 241-250	A*7401	1.6
B*490101,	GPC 240-259	**QRTRDIYISR**	**GPC 247-256**	A*3402	**0.59**
C*070102,	GPC 240-259	**RTRDIYISRR**	**GPC 248-257**	A*7401	**0.09**
C*160101)	GPC 240-259	TRDIYISRRL	GPC 249-258	B*4901	6.1
	NP 521-569	HCALMDCIMF	NP 528-537	B*3501	1.4
	NP 521-569	TSVLRAVLPR	NP 547-556	A*3402	1.9
	NP 521-569	RAVLPRDMVF	NP 551-560	C*1601	0.36
	NP 521-569	**AVLPRDMVFR**	**NP 552-561**	A*7401	**0.08**
	NP 521-569	**DMVFRTSTPR**	**NP 557-566**	A*3402	**1.6**
	NP 521-569	**MVFRTSTPRV**	**NP 558-567**	A*3402	**3.9**
	NP 521-569	VFRTSTPRVV	NP 559-568	C*0701	1.7
	NP 521-569	FRTSTPRVVL	NP 560-569	C*0701	0.69
8397490	GPC 52-82	GRSCTTSLYK	GPC 54-63	A*0301	0.83
	GPC 52-82	**SLYKGVYEL**	**GPC 60-68**	A*0201	**0.3**
	GPC 52-82	GVYELQTLEL	GPC 64-73	C*1601	1.6
(A*020101,	GPC 52-82	LNMETLNMTM	GPC 73-82	C*1601	6.3
A*030101,	GPC 240-343	YISRRLLGTF	GPC 253-262	C*1601	4.3
B*350101,	GPC 240-343	RLLGTFTWTL	GPC 257-266	A*0201	0.15
B*450101,	GPC 240-343	RWMLIEAELK	GPC 282-291	A*0301	1.18
C*040101,	GPC 240-343	AELKCFGNTA	GPC 288-297	B*4501	0.11
C*160101)	GPC 240-343	RLFDFNKQAI	GPC 314-323	A*0201	1.19
	GPC 240-343	AQMSIQLINK	GPC 330-339	A*0301	0.29
	GPC 412-451	YMERQGKTPL	GPC 419-428	C*1601	4.1
	GPC 412-451	GLVDLFVFST	GPC 429-438	A*0201	1.6
	GPC 412-451	LFVFSTSFYL	GPC 433-442	A*0201	3.54
	GPC 412-451	FVFSTSFYLI	GPC 434-443	A*0201	0.58
	GPC 412-451	FSTSFYLISI	GPC 436-445	A*0201	1.65
	GPC 412-451	STSFYLISIF	GPC 437-446	C*1601	6.0
	GPC 412-451	FYLISIFLHL	GPC 440-449	A*0201	1.54
	GPC 412-451	YLISIFLHLV	GPC 441-450	A*0201	0.12
	GPC 412-451	LISIFLHLVK	GPC 442-451	A*0301	0.45
	NP 344-377	LQSAGFTAGL	NP 356-364	C*1601	5.8
	NP 344-377	**SAGFTAGLTY**	**NP 358-367**	C*1601	**1.2**
	NP 344-377	FTAGLTYSQL	NP 361-370	C*1601	3.0
	NP 344-377	LTYSQLMTLK	NP 365-374	A*0301	0.11

a Peptides in boldface indicate positive epitopes.

To further examine the presence of shared peptide epitopes between Sierra Leonean and Nigerian survivors and to identify smaller epitopes within these regions, we utilized major histocompatibility complex class I (MHC-I)-binding prediction algorithms hosted by the Immune Epitope Database (www.iedb.org) to identify putative epitopes for further testing. To reduce the number of peptide candidates and to limit potential false-positive epitopes that might be generated if we were to use entire LASV NP or GPC sequences, we restricted this analysis to the deduced epitopes identified earlier that were specific for HLA expression of each individual. We tested peptides with <2.0 percentile rank based on predicted affinity unless there were less than three candidates under 2.0. In such cases, we tested at least the top three candidates regardless of percentile rank (Table 2). We stimulated autologous PBMCs from the survivors with 10-aa peptide epitope candidates for 5 h, followed by staining and flow cytometry analysis of IFN-γ and TNF-α expression by CD8^+^ T cells.

**FIG 3:**
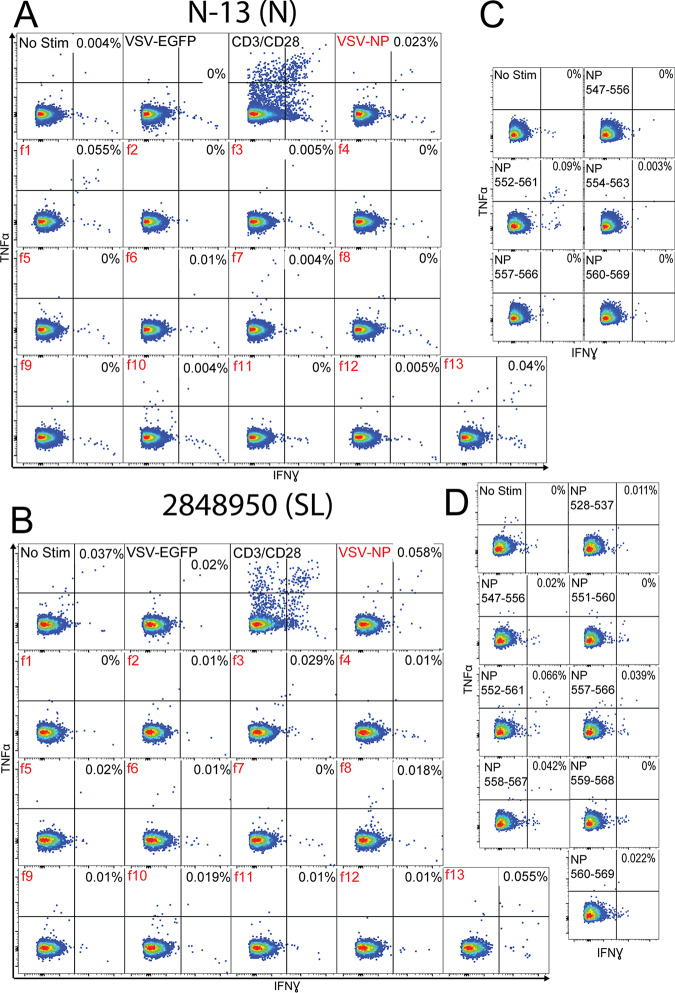
CD8^+^ T cell responses to NP antigens from a Nigerian (N-13) and Sierra Leonean (2848950) LF survivor. PBMCs from N-13 (A) and 2848950 (B) were incubated with rscVSVs encoding LASV NP and NP fragments (designated by an “f”) and evaluated by intracellular staining of IFN-γ and TNF-α flow cytometry. The ability of T cells to produce IFN-γ and TNF-α after engagement of the TCR was evaluated by incubation with antibodies against CD3 and CD28. Candidate peptide epitopes were identified by *in silico* MHC-1 prediction algorithms using reactivity to NP fragments and HLAs expressed by each LF survivor. The top predicted peptide epitopes were incubated with PBMCs from N-13 (C) and 2848950 (D) for 5 h in the presence of brefeldin A, and CD3^+^ CD8^+^ cells were evaluated by intracellular staining of IFN-γ and TNF-α flow cytometry.

Nigerian LF survivor N-13 and Sierra Leonean LF survivor 2848950 both responded to NP fragment 13 (f13, NP_508-569_), while no response was observed to rscVSVs encoding the adjacent fragment 12 (f12, NP_457-527_) (Fig. 3A and B), implicating the region encompassed by NP_521-569_ as containing a minimal epitope. The HLAs expressed by N-13 and 2848950 were predicted to bind five and eight peptides, respectively, within NP_521-569_. We incubated these peptides with PBMCs from each survivor and found that CD8^+^ T cells responded to epitope NP_552-561_ in both survivors (Fig. 3C and 3D). *In silico* MHC-1-binding prediction ranked HLA-A*74:01 binding to NP_552-561_ highest (NetMHCpan [cbs.dtu.dk/services/NetMHCpan/]; percentile rank, 0.09) among HLA alleles expressed by these two individuals. HLA-A*74:01 is the only allele shared by these two LF survivors and is the most likely restriction for NP_552-561_.

Survivor 2848950 also responded to NP_557-566_ and NP_558-567_. CD8^+^ T cell responses to NP_557-566_ and NP_558-567_ were similar (0.039% and 0.042% of CD8^+^ T cells, respectively) (Fig. 3D). This result strongly suggests that these T cells may respond to a smaller 9-aa epitope, NP_558-556_, although this peptide was not tested due to lack of additional PBMC samples.

**FIG 4:**
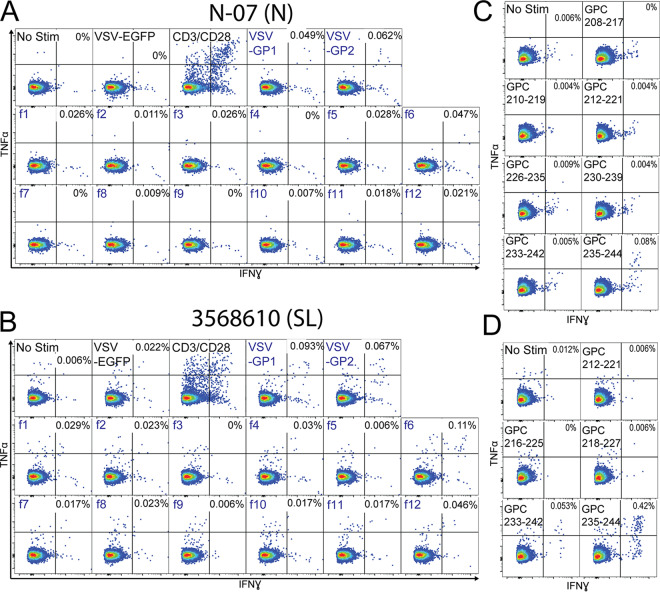
CD8^+^ T cell responses to GPC antigens from a Nigerian (N-07) and Sierra Leonean (3568610) LF survivor. PBMCs from N-07 (A) and 3568610 (B) were incubated with rscVSVs encoding LASV GPC and GPC fragments (designated by an “f”) and evaluated by intracellular staining of IFN-γ and TNF-α flow cytometry. Candidate peptide epitopes were identified as explained in Fig. 2. The top predicted peptide epitopes were incubated with PBMCs from N-07 (C) and 3568610 (D) for 5 h in the presence of brefeldin A, and CD3^+^ CD8^+^ cells were evaluated by intracellular staining of IFN-γ and TNF-α flow cytometry.

We also interrogated the carboxy terminus of GP1 based on the conservation of deduced epitope regions between Nigerian and Sierra Leonean survivors. CD8^+^ T cells from Nigerian survivor N-07 and Sierra Leonean survivor 3568610 coexpressed IFN-γ and TNF-α in response to rscVSVs encoding GPC f6 (GPC_194-256_) but not to surrounding fragments f5 (GPC_153-212_) or f7 (GPC_240-299_), suggesting the existence of an epitope within GPC_206-246_ (Fig. 4A and B). We tested the top seven putative peptide epitopes using cells from N-07 and top five putative peptide epitopes using cells from 3568610 and found strong responses to GPC_235-244_ in both individuals (Fig. 4C and D), with a weaker response to GPC_233-242_ only in 3568610 (Fig. 4D). Though both individuals have CD8^+^ T cell responses to GPC_235-244_, they do not share a class I allele. HLA-B*81:01 (expressed by N-07) and HLA-B*07:06 (expressed by 3568610) were predicted as having the highest affinity for GPC_235-244_ (NetMHCpan; percentile ranking of 0.03 for both alleles).

**FIG 5:**
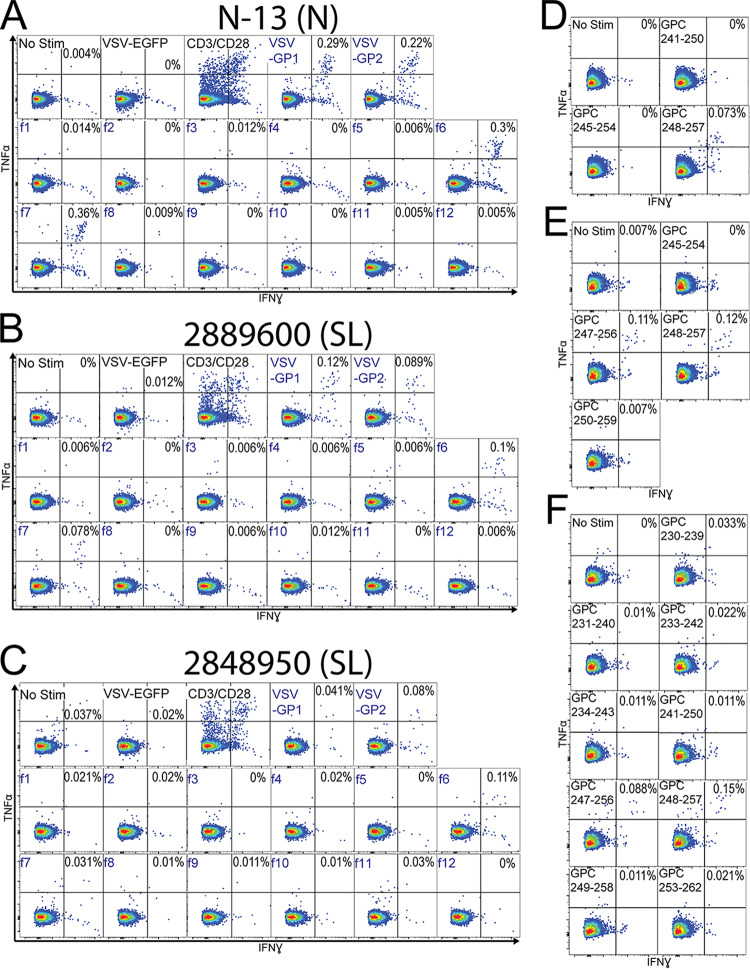
CD8^+^ T cell responses to GPC antigens from a Nigerian (N-13) and two Sierra Leonean (2889600, 2848950) LF survivors. PBMCs from N-13 (A), 2889600 (B), and 2848950 (C) were incubated with rscVSVs encoding LASV GPC and GPC fragments (designated by an “f”) and evaluated by intracellular staining of IFN-γ and TNF-α flow cytometry. Candidate peptide epitopes were identified as explained in Fig. 2. The top predicted peptide epitopes were incubated with PBMCs from N-13 (D), 2889600 (E), and 2848950 (F) for 5 h in the presence of brefeldin A, and CD3^+^ CD8^+^ cells were evaluated by intracellular staining of IFN-γ and TNF-α flow cytometry.

CD8^+^ T cells from Nigerian survivor N-13 and Sierra Leonean survivors 2889600 and 2848950 responded to rscVSVs encoding GPC f6 (GPC_194-256_) and f7 (GPC_240-299_) (Fig. 5A to C). Responses to GPC f6 and f7 were comparable in CD8^+^ T cells from N-13 and 2889600 (Fig. 5A and B), while 2848950 responded robustly only to GPC f6 (Fig. 5C). A weak CD8^+^ T cell response to f7 from survivor 2848950 (Fig. 5C) led us to test nine peptides derived from a larger region (GPC_206-259_), whereas the pool of peptide epitope candidates tested in PBMCs from N-13 (three peptides) and 2889600 (four peptides) were limited to sequences within the deduced epitope (GPC_240-259_). All three LF survivors responded to GPC_248-257_, while 2889600 and 2848950 also responded to GPC_247-256_ (Fig. 5D to F). Similar T cell responses to GPC_248-257_ and GPC_247-256_ (Fig. 5E and F) suggested these responses were to the single epitope GPC_248-256_, but we did not test directly this peptide epitope candidate due to lack of PBMC samples. No common HLAs were shared among all three individuals. However, N-13 and 2848950 both express HLA-A*74:01:01, while 2889600 and 2848950 share HLA-A*34:02:01. HLA-A*74:01:01 and HLA-A*34:02:01 are the top-ranked alleles predicted to bind GPC_248-257_ (NetMHCpan; percentile ranks of 0.09 and 0.55, respectively).

To assess if epitopes common between Nigerian survivors, NP_552-561_, GPC_235-244_, and GPC_248-257_ were more conserved than areas outside these regions, we analyzed the extent of amino acid conservation using 600 LASV NP and GPC sequences (420 from lineage II, 49 from lineage III, and 131 from lineage IV). We found a high degree of amino acid conservation within epitopes shared by Nigerian and Sierra Leonean survivors (NP_552-561_, GPC_235-244_, and GPC_248-257_) compared to nonepitope protein regions (99.30% versus 94.66%, *P* = 0.0193, two-tailed Mann-Whitney test). Individually, amino acid conservation is 99.5% for NP_552-561_, 99.4% for GPC_235-244_, and 99.0% for GPC_248-257_.

**FIG 6:**
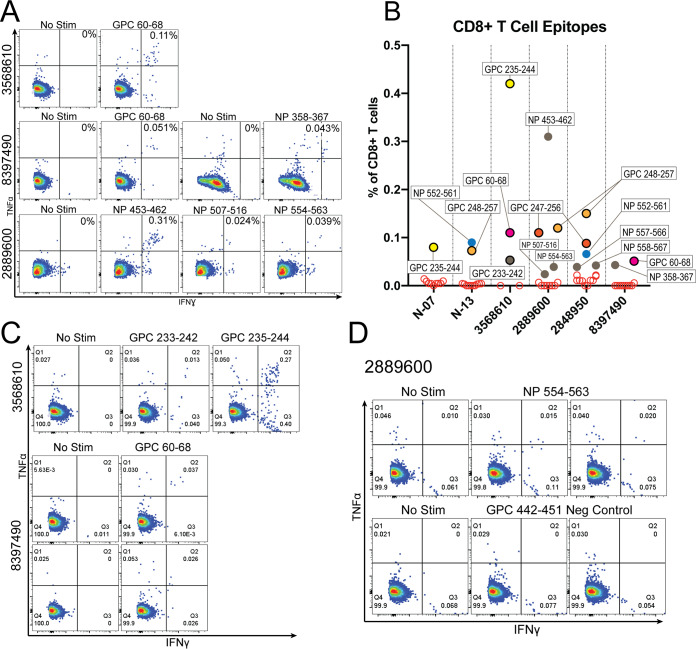
An additional seven 10-aa peptide epitopes discovered from LF survivors. (A) Candidate peptide epitopes were identified through *in silico* prediction of MHC-1 binding using reactivity to NP or GPC fragments and HLAs expressed by each LF survivor. Unstimulated and positive epitopes are shown. Epitopes were considered positive if we observed ≥3 events double positive for IFN-γ and TNF-α over the unstimulated controls. (B) Graph depicting percentages of CD3^+^ CD8^+^ cells double positive for IFN-γ and TNF-α in response to all candidate epitopes tested for each individual. Red empty circles indicate negative epitopes. Data from each patient are from a single experiment. GPC epitopes are denoted by a circle with a black outline, while NP epitopes are not outlined. Common epitopes are denoted by common colors, while epitopes only found in a single individual are gray. (C) CD8^+^ T cell responses to epitopes GPC_233-242_, GPC_235-244_, and GPC_60-68_ from independent experiments. (D) CD8^+^ T cell responses in duplicate from LF survivor 2889600 to a positive epitope, NP_554-563_, and a negative epitope, GPC_442-451_.

In addition to epitopes shared between Nigerian and Sierra Leonean LF survivors, we found an additional five 10-aa peptide epitopes (Fig. 6) using the methodology described above. We observed robust CD8^+^ T cell responses to T cell epitopes derived from NP and GPC sequences, with the greatest responses to NP_155-164_, NP_453-462_, and GPC_235-244_ (Fig. 6A to C). We also found CD8^+^ T cell responses from two LF survivors to a previously described HLA-A2-restriced epitope, GPC_60-68_ (Fig. 6A to C) (17). Both patients (3568610 and 8397490) with CD8^+^ T cell responses to this epitope express the HLA-A*02:01 allele. The same report (17) identified GPC_441-449_ as an HLA-A2-restricted epitope. While CD8^+^ T cells from 5513520 responded to a similar epitope, GPC_440-449_, this LF survivor does not harbor the HLA-A*02:01 allele, suggesting this epitope has multiple restrictions.

## DISCUSSION

We identified 11 peptide epitopes that produced CD8^+^ T cell responses from six LF survivors in both Nigeria and Sierra Leone. To our knowledge, only two of these epitopes have been previously described (2, 17), and this is the first study to identify CD8^+^ T cell epitopes from human LF survivors. Because we measured responses to whole LASV antigens, the epitopes can be placed in the context of both other peptide epitopes in the same individual as well as in the context of responses to the major antigenic proteins, NP and GPC, thereby revealing the comparative dominance of responses in each individual. We have also used this approach to identify T cell epitopes from Ebola survivors in Sierra Leone (16).

By delivering antigens through viral infection, the important biological mechanisms of antigen processing and presentation are maintained. Retaining these processes helps reduce the potential for cross-reactive T cells that were not generated or expanded during LASV infection. We were especially concerned about this possibility, as T cell responses are shaped early against a variety of pathogens in the antigen-rich environments of developing countries (18, 19). To reduce potential false-positive responses, we delivered LASV antigens through infection with rscVSVs to limit the number of peptides from a particular antigen that would naturally be presented by an antigen-presenting cell (20–22), thereby limiting potential false-positive responses. Other approaches, including the use of overlapping peptide pools, have been successfully used to identify T cell epitopes from a number of different infections (17, 23–26). However, this approach typically necessitates large numbers of PBMCs and subsequent rigorous validation. We were able to identify peptides epitopes from individuals with only 30 ml of blood, a limit imposed by our human subjects-approved protocol.

Due to these sample restrictions, we were unable to perform biological replicates in our experiments and relied on consistency of CD8^+^ T cell responses to rscVSVs encoding whole antigens and ∼60-aa fragments and to peptide epitopes. To reduce the possibility of identifying false epitopes, we instituted a robust criterion for positive epitopes, namely, 1.2-log increase in both IFN-γ and TNF-α expression instead of using increases in single cytokine expression. Weaker LASV-specific T cell responses or responses that resulted in the expression of a single cytokine may have been missed.

We had identified regions within GPC and NP that were more likely to harbor CD8^+^ T cell epitopes from Nigerian and Sierra Leonean LF survivors. We expected to find many common epitopes between these two groups of LF survivors. Instead, we found only a few smaller epitope regions in common (8). We were also surprised to find a lack of common HLA alleles among individuals with responses to the T-cell epitopes reported in the present study. No common alleles were found between individuals whose CD8^+^ T cells responded to GPC_235-244_. Similarly, no single HLA allele was found in common between all three survivors whose T cells responded to GPC_248-256_, although two alleles predicted to bind the epitope were found in two of the three responders. We also observed a diverse set of individual-specific epitopes.

The LASV genome only encodes four proteins, two of which typically harbor the majority of dominant T cell epitopes (27–32). Consequently, we, and others, have suggested that common epitopes could be encoded sequentially like “beads on a string” to maximize T cell reactivity while minimizing coding length (17, 33, 34). Because of our observations of highly immunogenic regions within the GPC and NP and the diversity of epitopes in these areas, it may be more prudent to design an immunogen that incorporates these highly immunogenic regions and not simply the smaller epitopes themselves. Importantly, we found these regions to be high conserved between lineages. This approach may lead to greater protection across populations with a diverse set of HLAs.

LASV epitope identification efforts have been focused on epitopes that may be presented by common HLAs (17, 35). Three LASV epitopes restricted to HLA-A*02, GPC_42-50_, GPC_60-68_, and GPC_441-449_ were identified through HLA binding and murine studies and shown to be protective in mice challenged with a recombinant vaccinia virus encoding LASV GPC (17). Two LF survivors in our study express HLA-A*02:01, and we found that the GPC_60-68_ peptide elicited responses in these individuals. Sierra Leonean LF survivor 8397490 responded to GPC f2 (GPC_40-98_), f11 (GPC_404-463_), and weakly to f1 (GPC_1-58_), which contain the previously identified epitopes GPC_60-68_, GPC_441-449_, and GPC_42-50_, respectively (REF). Sierra Leonean LF survivor 3568610, who also responded to GPC_60-68_, did not respond to GPC f2 or f11 and only very weakly to f1. This result could indicate a lack of sensitivity of our assay, a subdominant response from these previously identified epitopes, a cross-reactive response of using the peptide alone, or a combination thereof.

Multiple factors are responsible for the selection and dominance of epitope-specific T cells and associated responses during acute viral infections, including (i) precursor frequency (36), (ii) strength and stability of peptide MHC binding to its cognate T cell receptor (TCR) (37), (iii) expression of the antigen (38, 39), (iv) the extent and manner in which these antigens are processed and presented (40), and (v) the contribution of amino acids flanking the anchoring residues (41). We identified 12 10-aa peptide epitopes based on responses to whole LASV antigens and ∼60-aa polypeptides delivered by rscVSVs. Several epitopes were common among LF survivors, but HLAs were not universally conserved among LF survivors with common epitopes. Our data indicate that even for viruses with low proteomic complexity, there may be substantial variability in epitope-specific responses among individuals. Therefore, to elicit broad CD8^+^ T cell responses in diverse populations across West Africa, vaccines should deliver either whole antigens or encode those regions within the NP and GPC that we have identified as regions that elicit LASV-specific responses in the majority of survivors studied.

## MATERIALS AND METHODS

### Subjects.

This study was approved by the following institutions: Human Subjects Committees of the Broad Institute, The Scripps Research Institute, Tulane University’s Human Research Protection Program, the Sierra Leone Ethics and Scientific Review Committee, ISTH Research and Ethics Committee, and the Oyo State Research Ethical Review Committee. All subjects have a documented history of Lassa fever. Because of the manner in which samples were coded at the time of collection, no demographic, disease, or virus sequence information is available for the Nigerian participants. Demographic information and time from disease onset to an analysis of memory responses for Sierra Leonean patients are listed in Table 3.

**TABLE 3:** Demographic information for LF survivors

Subject	Age at infection	Sex	Yrs from infection to convalescent blood draw
N-07^*a*^	Unknown	Unknown	Unknown
N-13^*a*^	Unknown	Unknown	Unknown
3568610	7	F	5.9
2889600	19	F	2.2
2848950	18	M	7.8
8397490	17	F	0.1

a Convalescent blood collected in 2013.

### Sample collection and PBMC isolation.

Blood was collected in EDTA vacutainers at the Kenema Government Hospital (Kenema, Sierra Leone) or the Irrua Specialist Teaching Hospital (Irrua, Nigeria). PBMC isolation was performed according to standard protocols. Briefly, blood was diluted three times with phosphate-buffered saline (PBS) and layered onto Lymphoprep (StemCell Technologies) or lymphocyte separation medium (MP Biochemicals) and centrifuged at 1,450 rpm without break. Buffy coat layer was isolated and washed twice with PBS before being resuspended in CryoStor CS10 (StemCell Technologies), slow frozen at −80°C for 3 h, and stored in a liquid nitrogen dry shipper for shipment to the United States for analysis.

### rscVSV preparation and usage.

Plasmids used to rescue rscVSVs expressing LASV antigens have been described (8, 42, 43). LASV sequences were based on the Josiah strain from lineage IV. rscVSVs were rescued and characterized as described (8). PBMCs from LF survivors were incubated with rscVSVs at a multiplicity of infection (MOI) of 15 in a U-bottom 96-well plate. After 4 h, brefeldin A (4 μg/ml) was added, and cells were incubated overnight (∼16 h) at 37°C in 5% CO_2_ before staining with the following antibodies: anti-human brilliant violet 421 CD4 (clone RPA-T4; BioLegend), fluorescein isothiocyanate (FITC) CD8a (clone HIT8a; BioLegend), anti-human PE TNF-α (BD Biosciences), PE/Cy7 IFN-γ (clone 4S.B3; BD Biosciences), and APC IL-2 (clone MQ1-17H12; BD Biosciences). BD fixation/permeabilization kit was used for intracellular stains. Positive controls were treated similarly except for incubation with unlabeled anti-human CD3 (clone OKT3; BioXCell; 60 μg/ml) and CD28 (clone 9.3; BioXCell; 20 μg/ml) instead of rscVSVs.

### Peptide stimulations.

Peptide stimulations were performed at a concentration of 10 μg/ml (unpurified; AnaSpec). After 1 h stimulation, brefeldin A (4 μg/ml) was added, and cultures were incubated for an additional 4 h at 37°C in 5% CO_2_.

### (i) Peptide-MHC-1 binding prediction.

Deduced epitope sequences and HLA profiles (Table 2) were entered into the MHC-I Binding Prediction tool (version 2013-02-22) at the IEDB website (www.iedb.org) to generate a list of putative epitopes. We used the recommended settings (consensus and NetMHCpan) and selected all 10-aa peptides below the 2% prediction ranking for testing.

### HLA typing.

As previously described (16), genomic DNA was extracted from 1 × 10^6^ PBMCs using the Quick DNA miniprep plus kit (Zymo Research) and quality assessed by electrophoretic separation on an 0.8% agarose gel. For long-range amplification and library preparation, we used the TruSight HLA version 2 sequencing panel (CareDx) followed by sequencing of barcoded libraries on the Illumina MiSeq platform. Lastly, TruSight HLA Assign software (version 2.1 RUO) was used to analyze results and compared with sequences stored in the International ImMunoGeneTics Information System/HLA database (version 3.37). A reference DNA sample, IHW09263, was used for each sequencing experiment.

### Data availability.

All epitopes, including longer deduced epitopes and 10-aa peptide epitopes along with associated HLA alleles, have been submitted to the Immune Epitope Database (www.iedb.org; submission IDs 1000831, 1000832, and 1000836). In addition, more information on our subjects, including HLAs, is stored online by the Center for Viral Systems Biology (www.cvisb.org).
